# Reviewers List

**DOI:** 10.1002/rmb2.12082

**Published:** 2018-01-16

The publication of invaluable papers in the *Reproductive Medicine and Biology* depends on the prompt, careful review of submitted manuscripts. We would like to thank the Editorial Board members, the Editorial Advisory Board members and the following experts for reviewing manuscripts from November 1, 2016 to October 31, 2017.

Araki, Yasuhisa

Asada, Yoshimasa

Baba, Tsuyoshi

Chiba, Koji

Choi, Bum‐Chae

Durairajanayagam, Damayanthi

Enatsu, Noritoshi

Fukuda, Aisaku

Fukuhara, Shinichiro

Fukui, Atsushi

Furui, Tatsuro

Goto, Maki

Greco, Ermanno

Hara, Ryoei

Hara, Tetsuaki

Hasegawa, Isao

Hirota, Yasushi

Ihara, Motomasa

Ishihara, Osamu

Iwasa, Takeshi

Iwasaki, Akira

Iwase, Akira

Iwata, Hisataka

Izawa, Masao

Izumiya, Chiaki

Kajihara, Takeshi

Kanasaki, Haruhiko

Kasai, Tsuyoshi

Katagiri, Yukiko

Kawamura, Kazuhiro

Kawano, Yasushi

Kimura, Fuminori

Kobayashi, Hideyuki

Komiya, Akira

Kondoh, Nobuyuki

Kumagai, Jin

Kuroda, Keiji

Kyono, Koichi

Lee, Sung Ki

Maekawa, Ryo

Makino, Kenichi

Masuda, Hirotaka

Matsubayashi, Hidehiko

Miyagawa, Yasushi

Mukaida, Tetsunori

Murase, Tetsuma

Nagano, Masashi

Nakatsuka, Mikiya

Nambo, Yasuo

Nasu, Kaei

Nishioka, Emiko

Noce, Toshiaki

Okada, Hidetaka

Okitsu, Osamu

Ono, Masanori

Ozawa, Nobuaki

Sato, Yukiyasu

Shimizu, Aya

Shimizu, Keiko

Shiotani, Masahide

Shirasawa, Hiromitsu

Sugaya, Ken

Sugino, Toshihisa

Suzuki, Tatsuya

Suzuki, Hiroshi

Suzuki, Hiroyoshi

Suzuki, Nao

Takahashi, Toshifumi

Takashima, Akiko

Takeshima, Teppei

Takeuchi, Kazuhiro

Tamura, Isao

Tamura, Midori

Taniguchi, Fuminori

Taniguchi, Hisanori

Tatsumi, Kenichi

Uchida, Hiroshi

Uchida, Sayaka

Wada‐Hiraike, Osamu

Yamada, Mitsutoshi

Yamamoto, Yuri

Yamashita, Yasuhisa

Yoshida, Atsumi

Yoshida, Hiroaki

Yoshino, Osamu

Youssef, Mohamed A. F.

Yumura, Yasushi

